# Examination of physical activity development in early childhood: protocol for a longitudinal cohort study of mother-toddler dyads

**DOI:** 10.1186/s12887-023-03910-9

**Published:** 2023-03-20

**Authors:** Sarah B. Welch, Kyle Honegger, Megan O’Brien, Selin Capan, Soyang Kwon

**Affiliations:** 1 Buehler Center for Health Policy and Economics, Feinberg School of Medicine, Northwestern University, Arthur J. Rubloff Building, 420 E. Superior St, IL 60611 Chicago, USA; 2 Department of Emergency Medicine, Feinberg School of Medicine, Northwestern University, Chicago, USA; 3 Ann & Robert H. Lurie Children’s Hospital of Chicago, Chicago, USA; 4 Department of Physical Medicine & Rehabilitation, Feinberg School of Medicine, Northwestern University, Chicago, USA; 5 Max Näder Center for Rehabilitation Technologies and Outcomes Research, Shirley Ryan AbilityLab, Chicago, USA; 6 Smith Child Health Outcomes, Research, and Evaluation Center, Stanley Manne Children’s Research Institute, Ann & Robert H. Lurie Children’s Hospital of Chicago, Chicago, USA; 7 Department of Pediatrics, Feinberg School of Medicine, Northwestern University, Chicago, USA

**Keywords:** Accelerometry, Physical activity, Machine learning, Parenting, Child development

## Abstract

**Background:**

Physical activity (PA) development in toddlers (age 1 and 2 years) is not well understood, partly because of a lack of analytic tools for accelerometer-based data processing that can accurately evaluate PA among toddlers. This has led to a knowledge gap regarding how parenting practices around PA, mothers’ PA level, mothers’ parenting stress, and child developmental and behavioral problems influence PA development in early childhood.

**Methods:**

The Child and Mother Physical Activity Study is a longitudinal study to observe PA development in toddlerhood and examine the influence of personal and parental characteristics on PA development. The study is designed to refine and validate an accelerometer-based machine learning algorithm for toddler activity recognition (Aim 1), apply the algorithm to compare the trajectories of toddler PA levels in males and females age 1–3 years (Aim 2), and explore the association between gross motor development and PA development in toddlerhood, as well as how parenting practices around PA, mothers’ PA, mothers’ parenting stress, and child developmental and behavioral problems are associated with toddlerhood PA development (Exploratory Aims 3a-c).

**Discussion:**

This study will be one of the first to use longitudinal data to validate a machine learning activity recognition algorithm and apply the algorithm to quantify free-living ambulatory movement in toddlers. The study findings will help fill a significant methodological gap in toddler PA measurement and expand the body of knowledge on the factors influencing early childhood PA development.

## Background

Physical activity (PA) has numerous health benefits in children, including cardiovascular health, bone health, and motor and cognitive development [1]. Given that physical inactivity habits start to develop before age 5 years and are sustained over time [2, 3], it is alarming that a substantial proportion of preschoolers (age 3 and 4 years) are not sufficiently physically active [4–6]. Moreover, some studies [4–6], but not all [7], have shown that at the preschool age, females are less physically active than males. To better understand when and how PA habits develop in childhood, research investigating PA in children needs to focus on earlier age groups. Yet PA research in toddlers (age 1 and 2 years) is currently limited [1, 8], primarily because of a lack of analytic tools, such as accelerometer-based data processing, that can accurately evaluate PA in this age group [9].

Early childhood is a critical period in which children acquire movement proficiency and rapidly develop a range of motor skills [10]. By age 1 and 2 years, most children are expected to have reached important milestones for PA, such as walking and throwing a ball. Gross motor skills are viewed as the building blocks and foundation of PA in early childhood [11]. Gross motor competence has been shown to be cross-sectionally associated with PA in early childhood [12–14], including among children 2 years of age [15]. However, the association between gross motor competence and PA, as well as their temporal relationship through early childhood, has rarely been prospectively examined.

Parents [16–20], particularly mothers [21], play a critical role in shaping PA behavior during toddlerhood [22]. Parenting practices around PA are associated with a greater amount of PA in school-age children and older [23, 24]. In early childhood, parental modeling has been highlighted as a significant factor in determining child PA [25]. Maternal mental health status, such as depressive symptoms or psychological distress, is also associated with obesity in young children with and without developmental delay [26, 27]. However, the influence of parenting practices around PA, mothers’ PA, mothers’ parenting stress, and child developmental and behavioral problems on PA development in early childhood remains unclear.

Filling these knowledge gaps requires the development and application of new methodological approaches to accelerometer data processing for toddlers due to the lack of analytic tools to measure PA for this age group. We conducted a pilot study applying machine learning to the analysis of raw acceleration signals of accelerometer data among toddlers [28]. This study demonstrated that the machine learning analytic approach holds enormous potential for toddler activity recognition, with 79% accuracy [28]. We also conducted a feasibility study [8], which demonstrated acceptable compliance among toddlers to wearing a hip accelerometer in free-living settings. Building on these two pilot studies, we designed the longitudinal Child and Mother Physical Activity Study (CAMPAS) to achieve the following specific aims:

1. Aim 1: To refine and validate an accelerometer-based algorithm for toddler activity recognition; Hypothesis: a newly developed algorithm will have higher accuracy than the *Albert* algorithm (79%) from our pilot study.
2. Aim 2: To compare the trajectory of PA levels from age 1 to 3 years between males and females; Hypothesis: males will show a greater increase in PA between the ages of 1 and 3 years compared to females.

To explore the extent to which mothers’ and children’s characteristics influence the PA trajectories evaluated in Aim 2, we also have three exploratory aims:

- Aim 3a: To examine the association between gross motor development and PA development in toddlerhood.
- Aim 3b: To examine whether parenting practices around PA and mothers’ PA are associated with PA development in toddlerhood.
- Aim 3c: To examine whether mothers’ parenting stress and child developmental and behavioral problems are associated with PA development in toddlerhood.

## Methods

### Study design

The CAMPAS is a longitudinal study to observe PA development in toddlerhood and examine the influences of personal and parental characteristics on PA development. We plan to recruit 124 children (10–15 months of age) and their mothers, and conduct five waves of assessments at 6-month intervals (at the approximate child chronological ages of 12, 18, 24, 30 and 36 months). For Aim 1, a subsample of the child cohort (*n* = 62) will complete a validation study in waves 1 to 4. This subsample will be selected to ensure that half of the sample is female (31 children) and at least a third (21 children) comprises children from a preterm follow-up clinic or a developmental and behavioral pediatric clinic. In the Aim 1 validation study, each child participant will wear an accelerometer on one hip to collect data for 20 min in each of four settings in waves 1–4 during which period their movements will also be video recorded. The matched accelerometer and annotated video data will be used to develop and validate our algorithm for toddler PA recognition. For Aims 2 and 3, each child participant will wear an accelerometer on one hip for 24 h a day for 7 days in each of the five waves. For Aim 3a, mothers will proxy-report child gross motor development in all five waves. For Aim 3b, mothers will report their own PA and parenting practices around PA in waves 1, 3, and 5. For Aim 3c, mothers will report their parenting stress and child developmental and behavioral problems in waves 2 and 4.

### Study setting

The CAMPAS will be conducted in the Chicago area in Illinois, USA. Chicago is home to diverse racial and ethnic groups: 33% non-Hispanic White, 29% non-Hispanic Black, 29% Hispanic, and 7% Asian [29]. Study sites for data collection will include two commercial child play spaces (one located in the city of Chicago and the other in a Chicago suburb), participants’ homes, and public parks.

### Participant eligibility criteria

The eligibility criteria for child participants are age 10 to 15 months at the baseline assessment, without cerebral palsy or other medical conditions precluding physical movement. Only one child per mother will be eligible. The eligibility criteria for mother participants are age 18 years or older, self-identifies as the participating child’s mother, can communicate in English or Spanish, and lives with the participating child at least 50% of the time.

### Participant recruitment

Mother–child dyads will be recruited during a 2-year period, primarily from two source populations: patients from various pediatric clinics and users of partnered community-based organizations/programs. Recruitment pediatric clinics will include a preterm follow-up clinic, a developmental and behavioral pediatric clinic, and multiple pediatric primary care sites. The preterm follow-up clinic serves patients who were discharged from the Neonatal Intensive Care Unit (NICU) or Cardiac Intensive Care Unit (CICU) to monitor their development until age 5 years. The developmental and behavioral pediatric clinic serves pediatric patients for diagnosis and treatment of developmental behavioral disorders. These clinics were selected to recruit a developmentally diverse sample (e.g., preterm births).

Partnered community organizations/programs will include a Chicago commercial child playroom, a suburban park district, and the Maternal Infant and Early Childhood Home Visiting (MIECHV) Program. MIECHV is a national effort to support pregnant people and parents with early childhood support to increase child health and well-being [30]. We conducted our pilot study [28] and a community-based PA intervention program with these organizations, and we will leverage these existing partnerships for the CAMPAS.

Recruitment flyers will be placed in clinics (e.g., waiting and exam rooms) and community partner facilities and posted on social media. Flyers will be given to home visitors to distribute to participants of the MIECHV program. The flyers will be created in both English and Spanish and include a QR code that will take interested adults directly to an online screening survey. A phone number will also be provided so individuals can call the research team directly to learn more and complete the screening survey by phone. After eligibility is established, a member of the research team will begin the consent process with the potential participant by providing consent documents for their review, answering any initial questions, and scheduling the first visit for completion of the consent process, enrollment, and the baseline assessment.

### Participant retention

Because the CAMPAS is a 2-year longitudinal study, participant retention is critical. Our retention goal is ≥ 90% for a subsequent assessment, anticipating a final (wave 5) retention rate of 66% (0.9 × 0.9 × 0.9 × 0.9) over the 2-year follow-up period. The study will employ multiple strategies to reach this retention goal. To build rapport, each participant will have an assigned primary research staff member. This staff member will seek to develop relationships with their assigned families by managing all contact and interaction with them including scheduling and conducting their study visits; engaging in regular communication through phone calls, short text messages, and emails; and sending the participants birthday cards. Data collection visits will occur every 6 months and communication reminders halfway between these visits will ensure that research staff are in contact with participants every 3 months. We will obtain the phone number and email of another contact person who may be reached (e.g., aunt, sister, cousin) in case the participant contact information changes. Finally, if a participant misses an assessment at the proposed age, we will continue to invite the participant to complete five assessments at any age between 10 and 40 months, if there is at least a 4-month interval between assessments. If a participant moves out of the area, we will offer remote assessment options, such as Zoom meetings, online surveys, and mailing an accelerometer package and other study materials. We will provide incremental compensation over waves ($40 at wave 1, $50 at wave 2, $60 at wave 3, $70 at wave 4, and $80 at wave 5; validation study participants (Aim 1) will receive an additional $10 for each of waves 1–4). Monthly retention goals will be set and monitored.

### Study assessments (Table 1)

**Table 1:** Assessment schedule

Instrument	Wave 1 (12 Mo)	Wave 2 (18 Mo)	Wave 3 (24 Mo)	Wave 4 (30 Mo)	Wave 5 (36 Mo)
Participant demographics	X		X		
Anthropometry (recumbent length/height)	X	X	X	X	X
Accelerometer and video recording (validation sub-sample; Aim 1)	X	X	X	X	
Accelerometer (7-day wear; Aims 2–3)	X	X	X	X	X
Ages & Stages Questionnaire (Aim 3a)	X	X	X	X	X
Preschooler Physical Activity Parenting Practices (Aim 3b)	X		X		X
Physical Activity Questionnaire for mothers (Aim 3b)	X		X		X
Parental Stress Scale (Aim 3c)		X		X	
Parenting Daily Hassles Scale (Aim 3c)		X		X	
Strengths and Difficulties Questionnaire (Aim 3c)		X		X	

#### Validation study for Aim 1

To further develop and validate our accelerometer-based activity recognition algorithm, we will longitudinally collect validation study data at waves 1–4 in four different settings where toddlers often spend their time: home, indoor play space, childcare class, and outdoor playground. Data collection from child participants must be completed at each of the four main locations in waves 1–4, but the data collection may be completed in any order. We will collect accelerometer data during automobile rides from a sub-sample (20 participants) to collect accelerometer signals in this location. We will not collect validation study data at wave 5 (age 36 months), because physical/motor development in late toddlerhood is slower than in early toddlerhood and because a validated algorithm for children 3 to 5 years of age (the *Trost* algorithm) already exists [31].

The subsample of child participants (*n* = 62) included in the validation study will wear an ActiGraph wGT3X-BT accelerometer (a range of ± 6* g;* sampling at 100 Hz; Pensacola, FL) on the hip. ActiGraph accelerometers are the most widely used activity monitor in pediatric PA research. The child participants will then be allowed to engage in natural free-living activities in the given setting (e.g., indoor play space) for 20 min, such as interacting with their caregiver, siblings, and/or environment. Simultaneously, their activities will be video recorded using a GoPro Hero10 Black camera.

#### Child physical activity measurement for Aims 2 and 3

To assess their PA, child participants will wear an ActiGraph wGT3X-BT accelerometer for 7 days, for 24 h a day, in each of waves 1–5. The accelerometer will be worn at the level of the anterior superior iliac spine, underneath or on top of clothes, and on the side of the body (right or left) chosen by participants or caregivers for comfort. We elected to place one accelerometer per participant because wearing multiple accelerometers increases participant burden and reduces compliance and because prior preschooler studies showed that combined hip and wrist data did not significantly improve the machine learning algorithm performance, compared to hip data only [31, 32]. Caregivers will be asked to complete a monitor on/off time and sleep time log sheet. After the 7-day wear is complete, parents/caregivers will be asked to schedule a time for accelerometer pickup or instructed to mail the study package using the pre-paid envelope. With the chosen ActiGraph settings, the expected battery life exceeds the requested 7-day wear period. To promote wear-time adherence, a research staff member will contact the family midway through the 7-day wear period to check that the child participant is wearing the accelerometer and to troubleshoot any issues they might be having. As research staff receive the returned accelerometers, they will download, visually inspect, and pre-analyze the accelerometer data using the ActiLife software [33] to determine whether there are sufficient non-zero (wear) data (defined as ≥ 500 min per day for ≥ 3 days). If not, participants will be asked to re-wear an accelerometer to meet the 3-day criteria.

#### Gross motor competency for Aim 3a

Gross motor skills will be assessed by mothers using the Ages & Stages Questionnaire gross motor subscale (6 items) at each of the 5 waves [34]. The Ages & Stages Questionnaires are tailored to child age; the appropriate age-corresponding questionnaire will be used at each study visit.

#### PA parenting practices and mothers’ PA for Aim 3b

PA parenting practices will be measured using the Preschooler Physical Activity Parenting Practices (PPAPP) questionnaire at child age 12, 24, and 36 months (waves 1, 3, and 5) [35]. We selected this preschooler tool because there is no validated tool for toddlers. The questionnaire contains 30 items organized into 5 subscales: 15 items for PA encouragement, 3 items for inactivity promotion, 5 items for psychological control, 3 items for screen time promotion, and 4 items for restriction for safety concerns. Mother respondents will be instructed to choose the best answer on a 5-point Likert response scale (1 = never, 2 = rarely, 3 = sometimes, 4 = often, and 5 = always). The responses will be coded as 1 to 5 points and a subscale score will be calculated by averaging response points.

To test the hypothesis that maternal PA behavior influences child PA behavior via maternal PA modeling, we will also collect data on mothers’ PA as a parenting practice. At child age 12, 24, and 36 months (waves 1, 3, and 5), mothers will complete a self-report PA questionnaire that asks about the weekly frequency and duration of walking and other moderate- and vigorous-intensity PA, adopted from the National Health and Nutrition Examination Survey (NHANES) PA questionnaire [36] and the Family Life, Activity, Sun, Health and Eating (FLASHE) Study PA questionnaire [37]. Mothers will also report their average step counts for the prior week and month from their personal fitness tracking device or smartphone application (e.g., Fitbit, Health app for iPhone, Google Fit for Android), if available.

#### Mothers’ parenting stress for Aim 3c

Parenting stress will be measured using the Parental Stress Scale (PSS) [38] and the Parenting Daily Hassles Scale (PDHS) questionnaires, completed by participant mothers at child ages 18 and 30 months (waves 2 and 4) [39]. The PSS is an 18-item self-report scale that measures the level of stress experienced by a parent in the past month, accounting for both positive (e.g., emotional benefits, personal development) and negative (e.g., demands on resources, restriction) aspects of parenting. Each item is rated on a 5-point Likert scale (1 = strongly disagree to 5 = strongly agree). The PDHS is a 20-item self-report scale for assessing the frequency and intensity of 20 experiences that can be a “hassle” to parents of young children in the past month (e.g., “continuously cleaning up messes of toys or food”). The frequency provides an *objective* marker of how often those events occur, while intensity indicates a *subjective* appraisal of how much those events “hassle” or affect a parent. The frequency will be rated on a 4-point scale (1 = rarely, 2 = sometimes, 3 = a lot, and 4 = constantly). Intensity will be rated on a 5-point scale (from 1 = low to 5 = high). The PDHS response will be calculated by summing the scores from the frequency scale and the intensity scale.

#### Child developmental and behavioral problems for Aim 3c

To assess child externalizing behavioral problems, we will use the Strengths and Difficulties Questionnaire [40]. At child ages 18 and 30 months (waves 2 and 4), mothers will complete the Conduct Problem (5 items) and Hyperactivity (5 items) subscales of the Strengths and Difficulties Questionnaire version modified for children 1 and 2 years of age [41]. We elected to measure child externalizing behavior problems from age 18 months because externalizing behaviors begin to increase at age 12–17 months [41]. We believe that these 10 items are sensitive enough to capture externalizing behavior problems, given that in a study using only 5 of the 10 items, we identified 33% of preschool-age children with externalizing behavior problems in a US representative sample [42]. Each item will be rated on a 3-point scale (0 = not true, 1 = somewhat true, and 2 = very true). The externalizing behavioral problem score will be calculated by summing the scores of the 10 items.

#### Other measurements

We will collect participant information on race/ethnicity, main caregiver/childcare attendance, household structure (e.g., number of siblings in household), mother’s employment, mother’s education, neighborhood characteristics, brief child health history, and other caregiver information at wave 1. Items likely to change will be updated at wave 3. Research staff trained in pediatric anthropometry assessment will measure child recumbent length and weight in each of the five waves.

### Data collection procedures

All communication with participants will occur, and all written materials will be available, in English or Spanish, depending on participant preference. At each study visit, a research team member will collect all scheduled demographic data, anthropometric measurements, and survey items, and distribute the accelerometer, two waist belts, an instruction sheet, a wear time log sheet, and a pre-paid envelope for accelerometer return. If the child is included in the subsample of 62 participants in the validation study (Aim 1), they will play for 20 min while wearing an accelerometer and while the research team member video tapes them. At the completion of all study visit activities, participants will be given their 7-day wear accelerometer, shown how to wear it on their waist and be given instructions to take home along with a wear-time log sheet. The log sheet will be used to report monitor on/off time and sleep time for the 7 days of accelerometer wear following the study visit. As research staff receive the returned accelerometers, they will download, visually inspect, and pre-analyze the accelerometer data using the ActiLife software to determine whether there are sufficient wear data, defined as ≥ 500 min per day and ≥ 3 days. If there are not sufficient data, research team members will ask the participant to re-wear the accelerometer for another week. All project data will be collected and stored in REDCap [43, 44].

### Data processing

#### Accelerometer and video data processing for Aim 1

Three-axis accelerometer raw data (g-force) will be extracted from the device using the ActiLife software. Accelerometer data will be segmented into non-overlapping sliding windows, based on multiple window sizes suggested in prior studies (e.g., 5, 10, and 15 s) [28, 31]. We will extract the engineered features from the *Albert* and *Trost* algorithms (e.g., percentiles, dominant frequencies, standard deviation in lag and lead windows, etc.) [28, 31, 32] and from angle analysis for posture estimation [45]. The angle analysis was added to reduce confusion between “sedentary” and “standing” and between “walking/running” and “carried,” as shown in our pilot study [28]. We will engineer additional features by examining the angle values (defined as incident accelerometer orientation in relation to the gravity vector) for posture estimation [45], using the pilot data.

Ground truth activity types will be labeled based on video data using Behavioral Observation Research Interactive Software (BORIS) [46]. The coding guidelines developed for the pilot study [28] will be updated as needed. We will also reference a list of the 23 activity types for preschoolers used for the development of the *Trost* algorithm [31, 47, 48]. Two research staff will independently label the first four participant videos. The coded data (activity class label and activity start/end times in seconds) will be compared between the two coders to identify any disagreements. To resolve disagreements, the research team will review the video images associated with the disagreements, discuss, come to consensus, and update the coding guidelines, as needed. Based on the updated guidelines, the coders will independently label the next four participant videos and interobserver reliability will be calculated. This reconciliation process will be repeated until the inter-observer reliability reaches ≥ 95%. The remaining video files will be divided among the two coders for coding. After coding is complete, 10% of the coded files will be randomly selected to calculate the final inter-observer reliability. Detailed activity types will be re-classified into the five activity classes of interest: “walking/running,” “non-walking/running PA,” “light PA,” “sedentary,” and “being carried by an adult.”

#### Accelerometer data processing for Aims 2 and 3

The 7-day raw accelerometer data will be extracted from the device using the ActiLife software. Non-wear and sleep periods will be identified. A non-wear period will be defined as ≥ 20 consecutive minutes of zero counts [49–51]. Participants with at least 500 min of wear per day and 3 wear-days will be included for analysis. A sleep period, defined based on mother-reported sleep onset (bedtime) and sleep offset (waking time), will be identified based on a sleep algorithm developed by Tracy et al. [52]. The wear time log sheet will also be used to cross-check non-wear and sleep periods.

### Data analyses

#### Aim 1 analysis

We will further develop our machine learning algorithm from our pilot study to classify PA in toddlers. Ground truth activities (from the video coders) will be synchronized with the accelerometer data based on detection of a specific movement pattern simultaneously acquired by camera and accelerometer. Data from the validation study will be split into a training set for model development and a testing set for model validation. A random forest classifier has been selected for primary development because it is a widely implemented ensemble-based classifier [31], showing the highest accuracy for activity recognition [28, 32, 48, 53]. Additional machine learning algorithms may be considered to maximize classification performance for the dataset.

##### Model training

A random forest classifier will be trained to classify the five activities (walking/running, non-walking/running PA, standing, sedentary, and being carried), using the feature set from the *Albert* [28] and the *Trost* algorithms [31]. Feature selection and dimensionality reduction techniques (e.g., principal component analysis, feature embedding) will be considered to minimize risk of overfitting. A grid search approach will be used to explore a range of window sizes (5, 10, and 15 s) [54] and hyperparameter values (e.g., the number of trees used in the random forest). From these combinations, the optimal set of parameters will be chosen based on cross-validated performance on a validation sample. A leave-one-subject-out (LOSO) cross-validation will be performed to assess overall classification accuracy. Precision, recall, F1 score, and confusion matrices of the classification will be examined to learn about the patterns of misclassifications and refine the model.

##### Model evaluation

Using the testing dataset, we will generate confusion matrices of the five-activity classification. Model performance (precision, recall, and F1 score) for activity classification will be estimated per participant and averaged to determine overall performance. We will evaluate the overall model performance using weighted kappa [47]. Next, we will evaluate the validity of the new algorithm for estimation of walking/running time (minutes) and overall PA time (the sum of walking/running time and non-walking/running PA time; minutes) against the ground truth data, using confusion matrices [55] as well as Bland Altman plots and root mean squared error. To explore whether the model performance differs by sex or age, we will repeat these analyses by sex assigned at birth and by age (1 year vs. 2 years of age).

#### Aim 2 and 3 analyses

To be included in the PA trajectory analysis (Aim 2), participants should complete at least three of the five waves of PA assessments. We will estimate daily walking/running time (minutes/day) and daily overall PA time (minutes/day) using the algorithm developed and validated in Aim 1. Multiple days of PA data per assessment wave will be averaged. As we hypothesize that individuals will present diverse start levels (intercept) and change rates (slope) of the outcomes, a growth curve model will be used to describe deviation or variation of individuals’ outcome values from the population growth curve. A growth curve model will be fit to depict the trajectory of daily walking/running time over age in months from age 12 to 36 months. A quadratic model will be initially fit to allow for a non-linear trajectory. The growth curve model analysis will be repeated for the daily overall PA time outcome. To examine whether PA trajectory differs by sex, the sex variable will be added as a predictor. The interaction of sex and age (sex*age) will also be considered. The model will also account for the season of assessment, distinguishing between winter months (December to March) vs. other seasons.

#### Aim 3a analysis

To examine the association of child PA with gross motor skills, we will expand the growth curve model fitted in Aim 2 and conduct mixed model analysis by adding gross motor skill scores as a predictor.

#### Aim 3b and 3c analyses

We will conduct bivariate analyses between child PA the potential correlates (parenting practices around PA, mothers’ PA, mothers’ parenting stress, and child behavioral problems). Any factors that are found to be statistically significantly associated with child PA in the bivariate analyses (*p* < 0.05) will be selected to be included in the subsequent multivariable mixed model analysis. The mixed linear regression model will predict child PA by the selected factors. The model will include within-subject random effects for repeated measures. In addition, the following covariates will be considered: mother’s racial and ethnic background, mother’s education level, mother’s employment status, the number of young children in the household, child’s childcare attendance, child sex, and child age.

### Sample size calculation

#### Sample size calculation for Aim 1

We determined a sample size for Aim 1 based on a prior study [47] that examined the validity of a machine learning activity recognition algorithm for preschool-age children. That study had a sample size of 31 children and collected data during a 20-minute free-play session. The study found that overall accuracy for a hip random forest classifier (15-s window) was 81% (95% confidence interval [CI] = 79–83%; width of CI = 4%), which was significantly higher than the accuracy of a wrist random forest classifier (75%; 95% CI = 73–77%). Given that the *Albert* algorithm from our pilot study showed 79% accuracy [28], we estimated that a sample size of 31 children would be sufficient to detect at least 4% accuracy improvement from 79% to ≥ 84%. As we will equally split the validation study data into a training set and a testing set, 62 participants (31 participants for training and 31 participants for testing) will be required for Aim 1.

#### Sample size calculation for Aims 2 and 3

Aim 2 and 3 statistical analyses will include participants who have at least three waves of accelerometer data. As wave 3 is predicted to have the smallest sample size among waves 1, 2, and 3 due to drop out, we calculated a required sample size to detect sex difference at wave 3. Based on our pilot study that showed 8 min less moderate and vigorous PA among females than males with a standard deviation (SD) of 14 min, 100 participants (50 males and 50 females) would be required to reject the null hypothesis of equal means when the population mean difference is 8 min with a SD for both groups of 14 min (effect size of 0.57) at 80% power and a significance level of 0.05 using a two-sided two-sample equal-variance t-test. Also, published recommendations regarding a sample size for growth curve modeling suggest that a sample size approaching at least 100 is preferred and at least 3 repeated measures per individual are typically required [56]. Therefore, our goal is to retain at least 100 participants at wave 3. Assuming a 90% retention at each subsequent wave or an 81% (0.9 × 0.9) retention rate at wave 3, we will need to recruit 124 participants (100 ÷ 0.81) at baseline.

## Discussion

To our knowledge, the CAMPAS will be one of the first studies to use longitudinal data to validate a machine learning activity recognition algorithm and apply the algorithm to quantify free-living ambulatory movement in toddlerhood. This study will help fill a significant methodological gap in toddler PA measurement and expand the body of knowledge in early childhood PA.

The algorithm that will be further developed and validated in the CAMPAS will improve upon current algorithms used for activity classification of young children. The *Albert* algorithm was developed in activity trials and, as such, its performance is expected to decline substantially when applied in free-living settings [57, 58]. Current algorithms, both cut-point and machine learning algorithms, are mostly based on laboratory data, which often exhibits a substantial reduction in accuracy (20–50% in adults [59–61] and 11–15% for preschoolers [48]) when implemented in free-living conditions. Model training and validation on free-living data is crucial because the algorithms will ultimately be utilized for analyzing free-living data. Recently, Ahmadi and Trost demonstrated that among preschool-age children, machine learning-based PA classification model showed substantially higher accuracy (weighted kappa = 0.72) than four cut-point methods (weighted kappa = 0.31 to 0.44) [47]. Therefore, a machine learning approach has the potential to perform more accurate toddler activity recognition than the traditional cut-point approach.

### Potential issues and limitations

While we have made every attempt to identify and prepare for practical or operational issues, we know that behavioral research including human subjects is inherently susceptible to deviations from the planned protocol. We may find that the new algorithm developed in Aim 1 does not perform better than the existing *Albert* algorithm or the *Trost* algorithm. In this case, we would use the existing algorithm to estimate walking/running time and overall PA time for Aims 2 and 3. Our approach of assigning a single activity to a window with multiple activities could cause classification error. To assess its impact on classifier performance, we will conduct sensitivity analyses to examine whether classifier performance differs with and without the mixed-activity windows.

Activity labeling is a time-consuming task and could pose a potential practical challenge. We will explore innovative ways to reduce the burden, for example, by implementing partially automated video-based labeling and unsupervised learning techniques [62]. Although we plan to collect data in four common free-living settings where most toddlers often spend their time, these settings still do not fully represent all free-living environments.

In Aims 2 and 3, some participants may drop out or have incomplete accelerometer data in some waves even after the imputation of incomplete accelerometer data. Growth curve modeling allows for missing data and provides valid results under the assumption of missing data at random where the missing-ness is “ignorable.” However, the possibility of non-ignorable missing-ness cannot be ruled out. Therefore, we will conduct sensitivity analyses using non-ignorable pattern-mixture and selection models to assess the robustness of our conclusions across these different models for missing data [63]. For Aim 2, even if we do not find sex differences in PA trajectory, gross motor skill and PA parenting practice data can still be utilized to examine the associations with PA trajectory in exploratory Aim 3.

In Aim 3c, mothers’ self-report of psychological stress is prone to measurement error. To complement the parenting stress measure, we will collect the objective markers of stressful events using the PDHS. Mothers’ self-reported PA is also a limitation. To complement mothers’ self-reported PA data, we will collect step count data recorded in the fitness tracking application of their smartphone.

Finally, we understand that sex, gender, identity, and primary caregiving role are neither static nor uniform, and that families come in many different configurations and not all of them include mothers. The focus on mothers and the exclusion of parents who do not identify as the child's mother is a consequence of the existing literature base, which specifically reports the influence of the mother on child PA. Further, the literature suggests that children and mothers bidirectionally influence each other’s PA behaviors. Mothers’ PA reduction is largely explained by lack of time and fatigue [64–66] due to new and increased stressors related to childcare [67, 68]. Parenting stress is particularly high among mothers of young children with externalizing behavior problems, which are known to peak at age 2–3 years [69] and present in one in three young children in the US [42]. Our study protocol recognizes that parents [16–20], particularly mothers [21], play a critical role in shaping child PA habits.

### Study implications

Our study will develop and validate a monitor-independent machine learning algorithm for toddler activity recognition using longitudinal free-living accelerometer data. Utilizing this algorithm, we will describe the PA trajectory from age 12 to 36 months, with a focus on sex differences in PA development during this period and the influence of mothers on PA behaviors in toddlers.

The output of this study will contribute to knowledge of PA development among males and females in early childhood (age 1 and 2 years) and the role that mothers play in influencing toddler PA practices. The new analytic tool developed and validated in Aim 1 will be made publicly available to facilitate dissemination and application by other researchers. Understanding the trajectory of toddler PA and the influence of mothers on PA behaviors during this time is critical for revealing new opportunities for program and policy interventions that will promote increased toddler PA. Ultimately, this study will provide necessary epidemiologic data that can contribute to establishing Physical Activity Guidelines for children under age 3 years.

## Data Availability

Not applicable. This manuscript does not present any data or study findings.

## Abbreviations

BORIS: Behavioral Observation Research Identification Software
CAMPAS: Child and Mother Physical Activity Study
CI: Confidence interval
CICU: Cardiac Intensive Care Unit
FLASHE: Family Life, Activity, Sun, Health and Eating study
LOSO: Leave-one-subject-out
MIECHV: Maternal Infant and Early Childhood Home Visiting
NHANES: National Health and Nutrition Examination Survey
NICU: Neonatal Intensive Care Unit
PA: Physical Activity
PDHS: Parenting Daily Hassles Scale
PPAPP: Preschooler Physical Activity Parenting Practices questionnaire
PSS: Parental Stress Scale
SD: Standard deviation
